# Supplementary material for: Physiological and pathogenic T cell autoreactivity converge in type 1 diabetes
Source: Nat Commun. 2024 Oct 29;15:9204. doi: 10.1038/s41467-024-53255-9 (PMC11522472; doi:10.1038/s41467-024-53255-9)
Supplement: Supplementary file 1 — Supplementary information [file 41467_2024_53255_MOESM1_ESM.pdf]

Supplementary information

Physiological and pathogenic autoreactivity converge in Type 1 Diabetes

Anne Eugster, Anna Lorenc, Martin Kotrulev, Yogesh Kamra, Manisha Goel, Katja Steinberg-Bains, Shereen Sabbah, Sevina Dietz, Ezio Bonifacio, Mark Peakman, Iria Gomez-Tourino

Supplementary figures

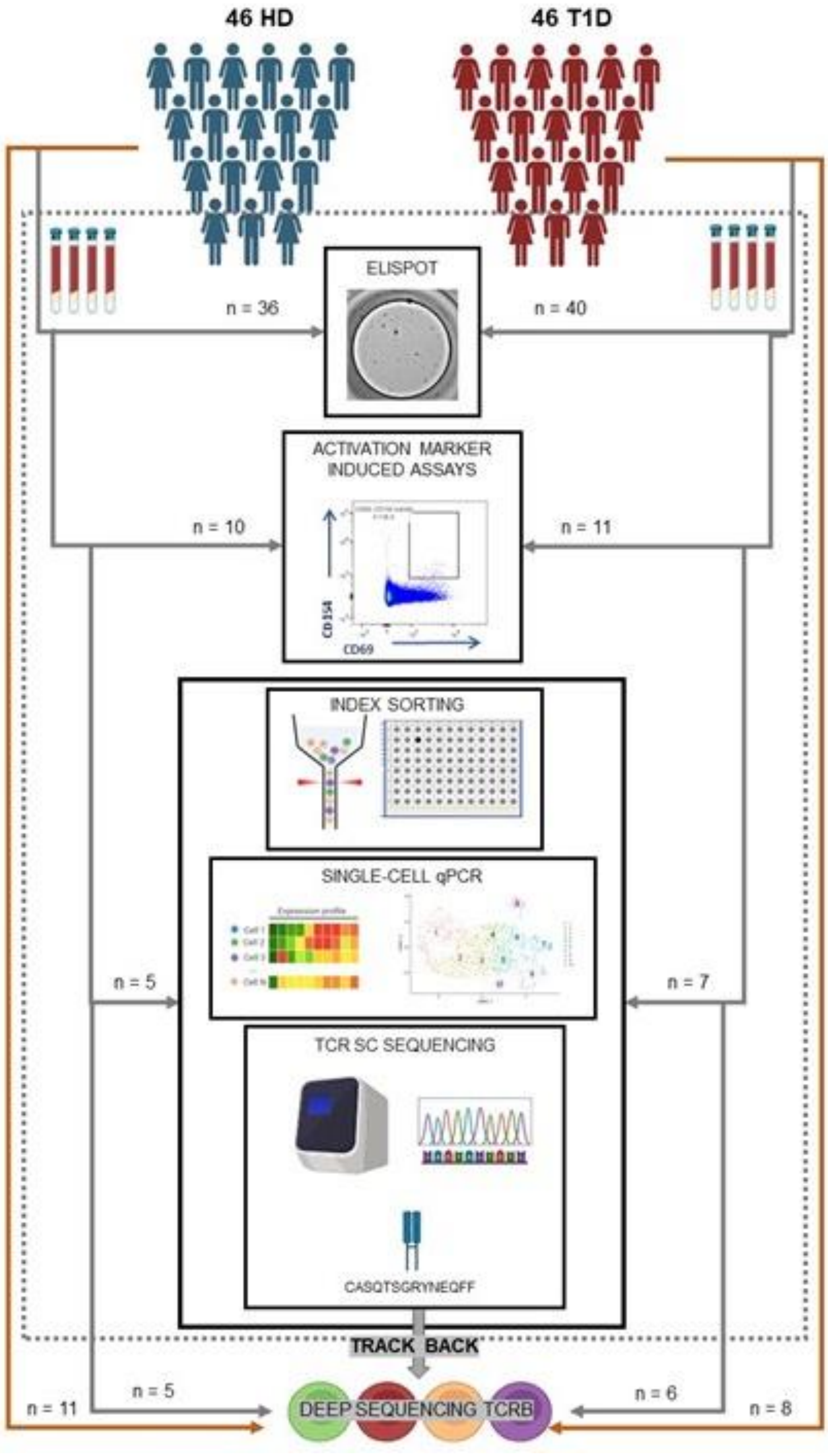

Supplementary Figure 1: workflow of the study. Some of the content has been created in BioRender. Gomez-tourino, I. (2024) BioRender.com/g37x166

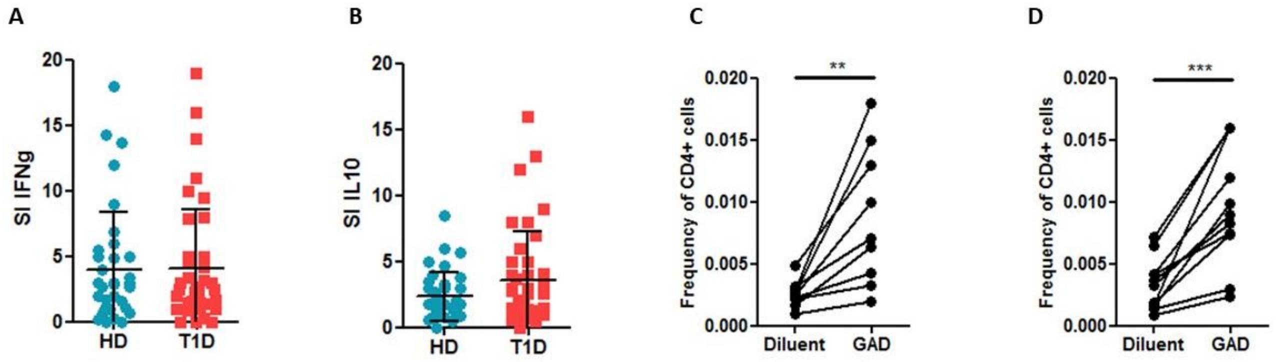

Supplementary Figure 2: ELISPOT and AIM assay results. A,B: PBMCs from 36 HD (blue) and 40 T1D patients (red) were stimulated with GAD6565. Shown are ELISPOT stimulation indexes (SI) for IFN-γ (**A**) and IL-10 (**B**). Two-sided Mann-Whitney U test (A: p=0.94. B: p=0.25). **C-D:** Frequency of $CD154^+$ $CD69^+$ cells for GAD65 *versus* GAD65 diluent in HD (**C**) and T1D patients (**D**) (two sided paired t test. C: p=0.0034. D: p=0.0002). **: p<0.01. ***: p<0.001. Error bars represent standard deviations.

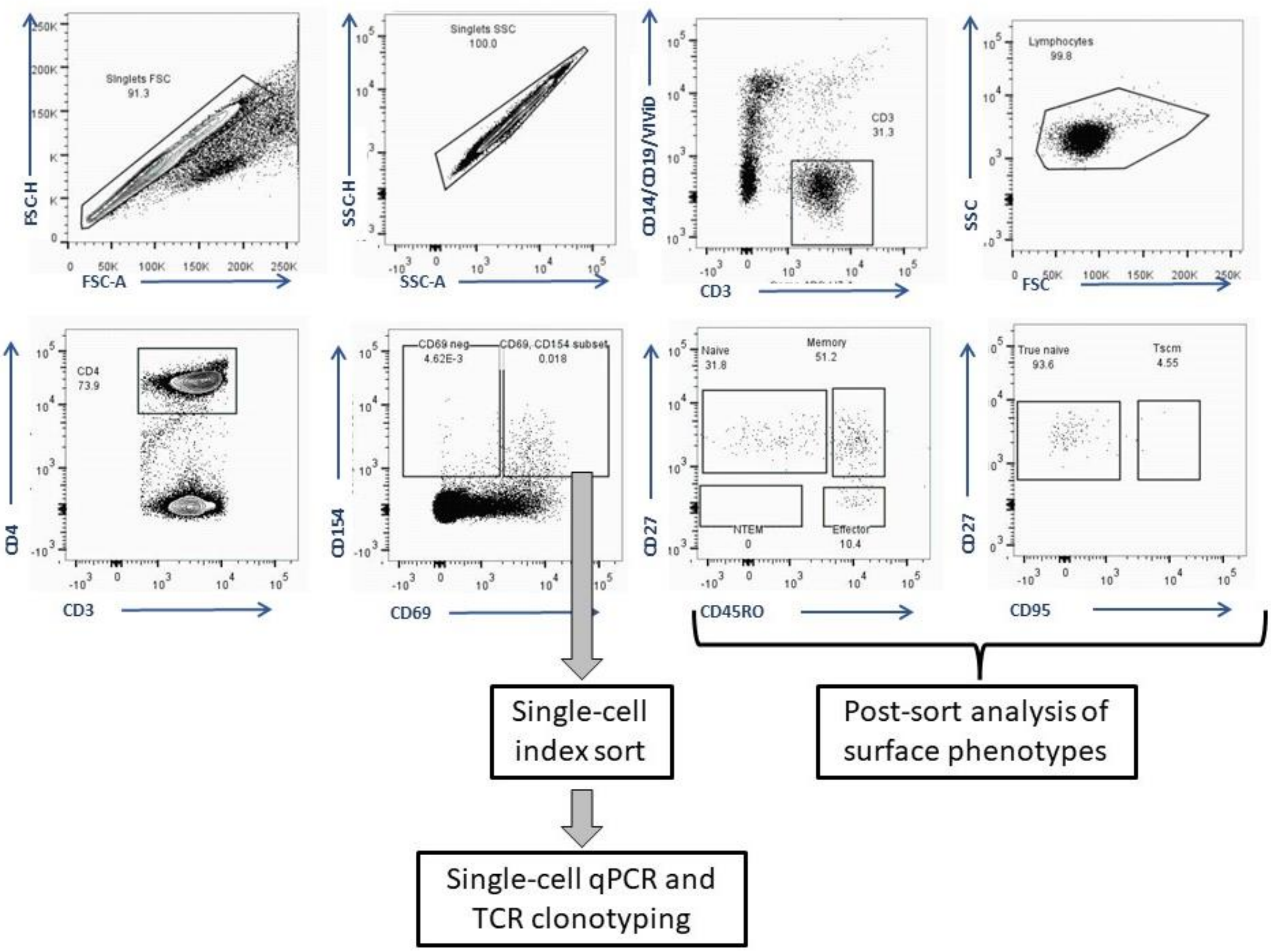

Supplementary Figure 3: Gating strategy for the AIM assay and single cell index sort. Fresh PBMCs were cultured with either GAD65 protein or GAD65 diluent, or CMV grade 2 antigen. Non-adherent cells were harvested after 18h, washed, stained, and live $CD3^+$ $CD4^+$ $CD154^+$ $CD69^+$ cells were index sorted. Plates were snap frozen in dry ice and stored until single-cell qPCR and TCR clonotyping. After sort, surface phenotype analysis took place for each index sorted cell, analyzing the expression levels of CD54RO, CD27 and CD95, and classifying each cell as true naïve (TN: $CD45R0^{neg}$, $CD27^+$, $CD95^{neg}$), central memory (CM: $CD45R0^+$, $CD27^+$), effector memory (EM: $CD45R0^+$, $CD27^{neg}$) non-terminated effector memory (NTEM: $CD45R0^{neg}$, $CD27^{neg}$), or stem cell-like memory (Tscm: $CD45R0^{neg}$ $CD27^+$ $CD95^+$)

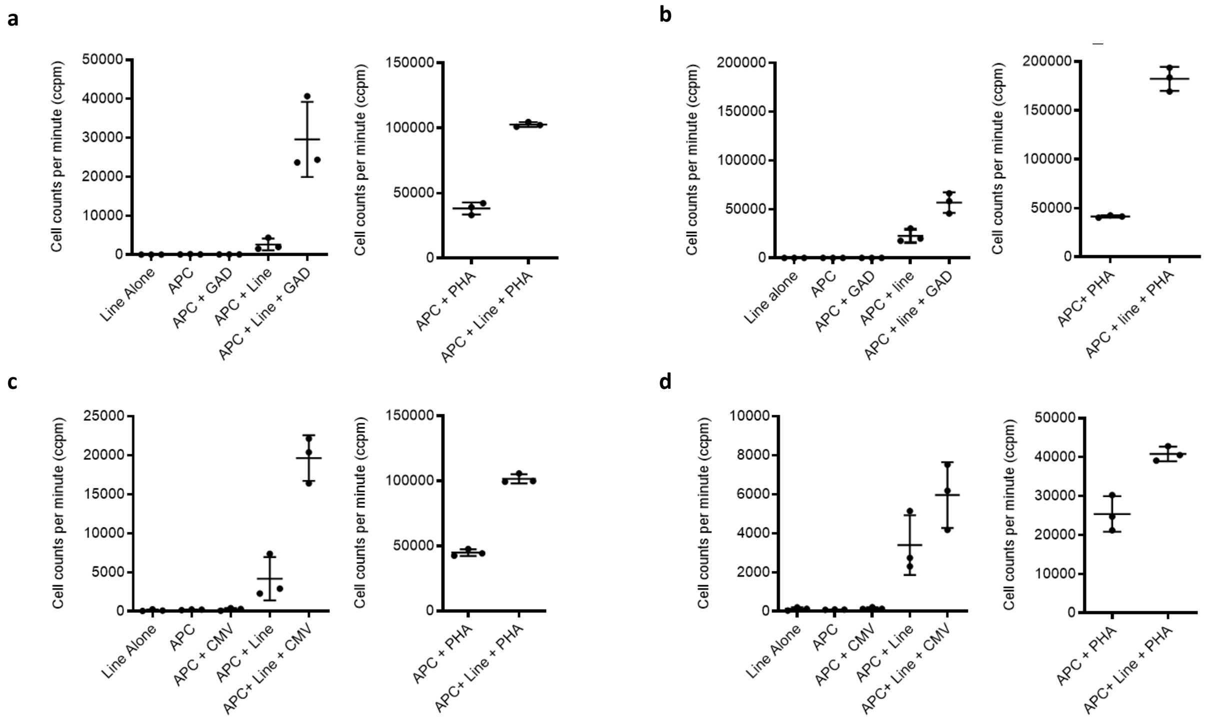

Supplementary Figure 4. Responsiveness of GAD65 and CMV cell lines. PBMCs were stimulated with either GAD65 or CMV extract for 18h, and live $CD3^+$ $CD4^+$ $CD154^+$ $CD69^+$ cells were bulk sorted into U-bottom 96-well plates containing irradiated fresh mixed feeders and PHA. Cells were restimulated after 12-14 days and subjected to proliferation assays with the cognate antigen. All conditions were run in triplicate and proliferation readings (CPM) averaged. APC: antigen presenting cell. PHA: phytohemagglutinin. Shown are means and standard deviations.

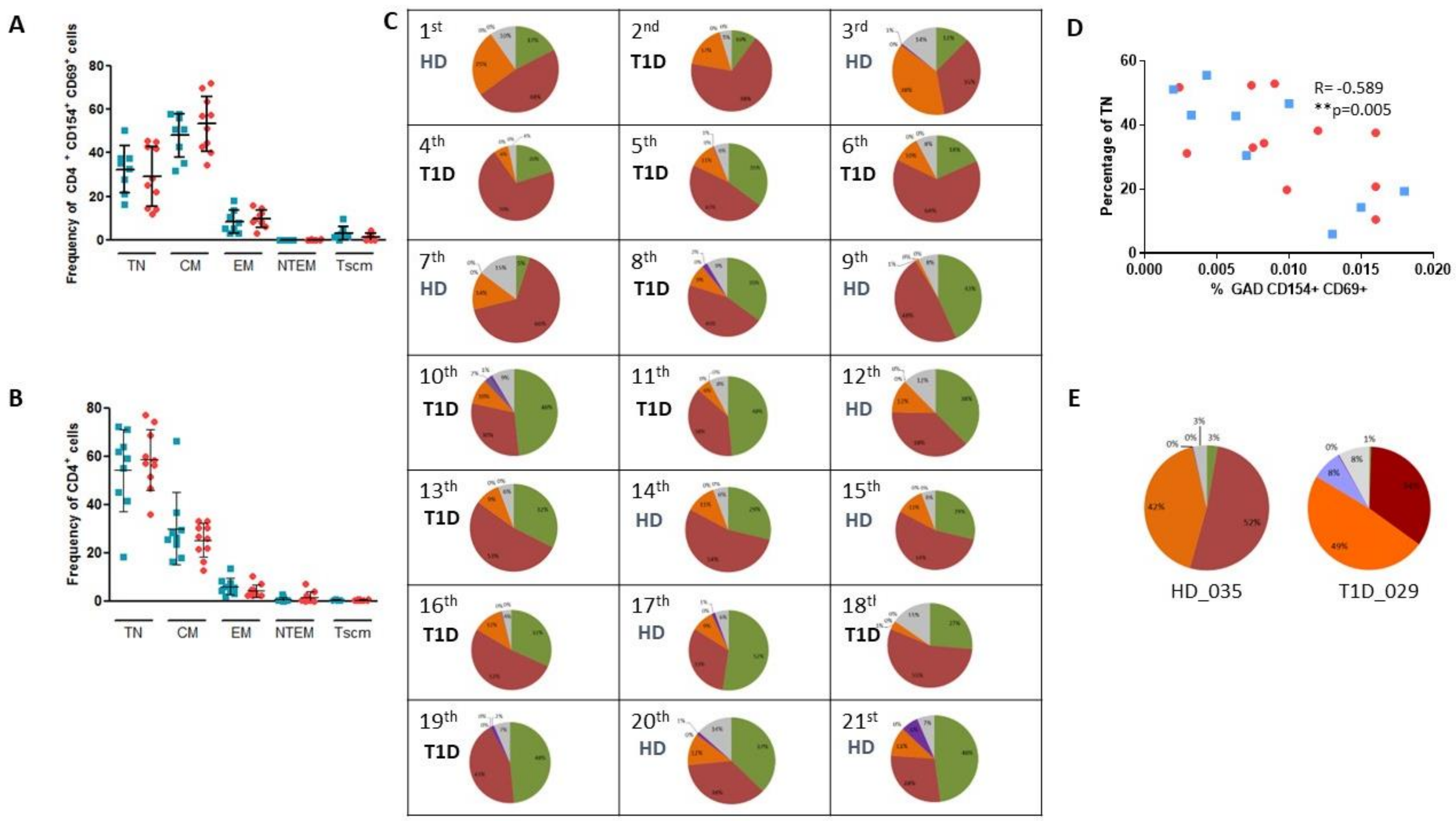

Supplementary Figure 5: Surface phenotype analysis of antigen-specific cells. A. Surface phenotype of SEB CD154⁺ CD69⁺ cells (two-sided Mann Whitney U test. p>0.05 in all instances). **B:** Surface phenotype of CD4⁺ cells not stimulated with antigen (same donors and blood draw as Figure 1D and Supplementary Figure 5A) (two-sided Mann Whitney U test. p>0.05 in all instances). **C.** Pie charts representing the phenotype pattern of GAD65-specific CD154⁺ CD69⁺ cells in each donor. Donors are sorted from higher lo lower frequencies of GAD65-specific cells. Green: TN. Red: CM. Orange: EM. Tscm: dark purple. NTEM: light purple. Grey: transitional cells (i.e. outside gates). **D:** Correlation between the total frequency of GAD65-specific cells and the portion of TN cells among them (Spearman correlation. Blue: HD. Red: T1D patients). **E:** Pie charts representing the phenotype pattern of CMV-specific CD154⁺ CD69⁺ cells for HD_035 and T1D_029. Error bars represent standard deviations.

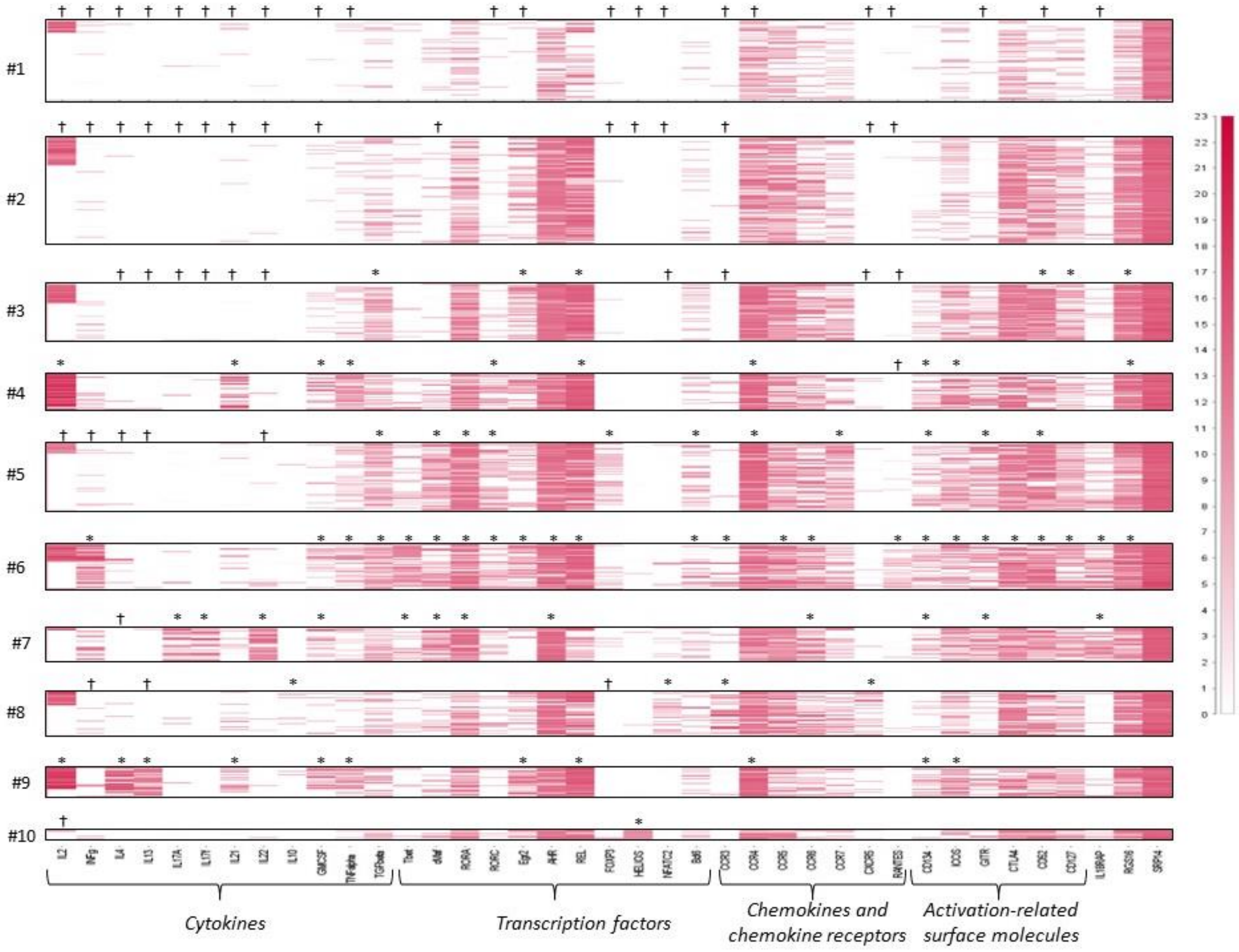

Supplementary Figure 6: Heatmap of GAD65-specific cells. Numbers on the left refer to genex clusters. Asterisks: significantly upregulated genes. Cross: significantly downregulated genes. Note that the actual p-values are shown in Supplementary Table 3 for simplicity.

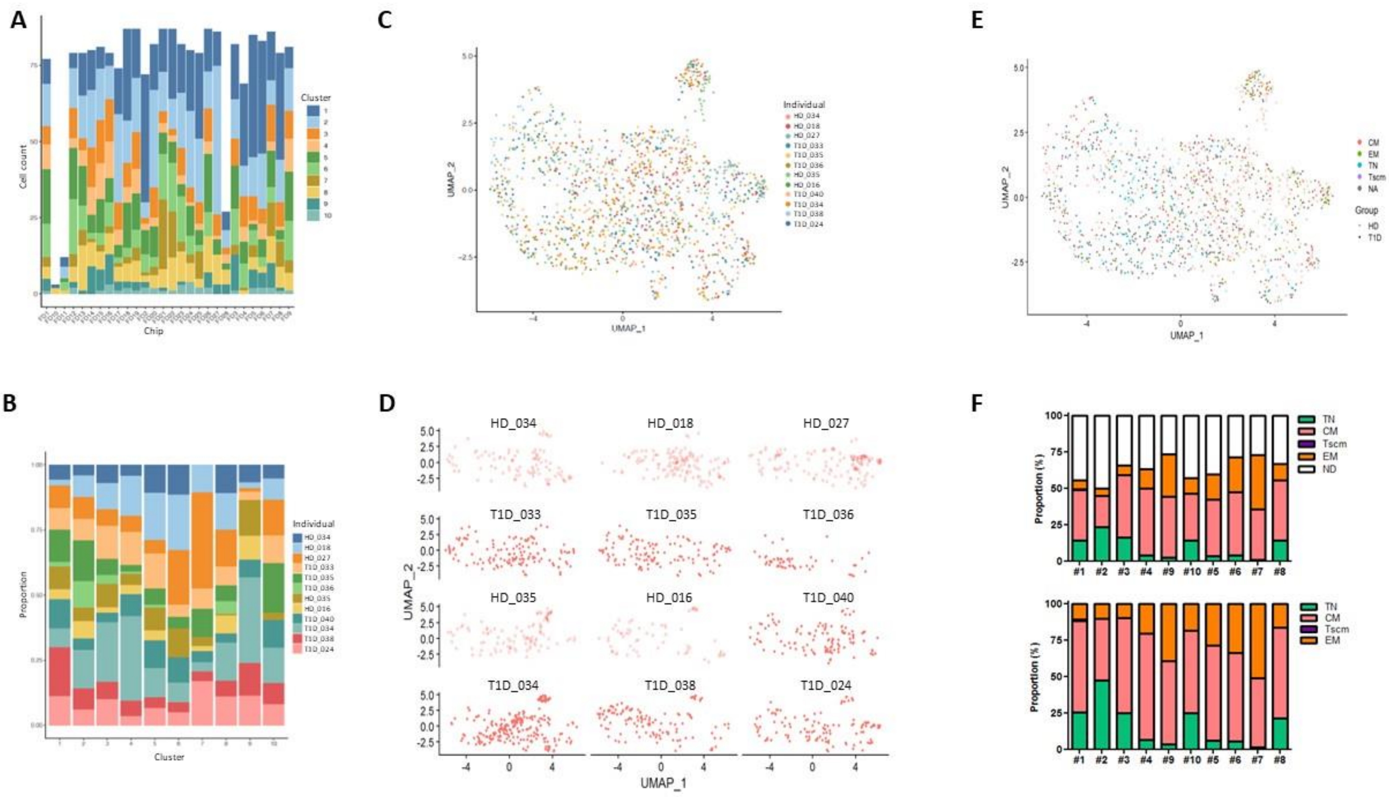

Supplementary Figure 7: A. Number of cells per genex cluster and qPCR chip. **B.** Proportion of cells of each donor in each genex cluster. **C.** UMAP plot coloured by donor. **D.** Individual UMAPs for each donor. **E:** UMAP plot coloured by surface phenotype. **F:** Surface phenotypes not normalized (top) and normalized to events with unassigned surface phenotypes (bottom).

a

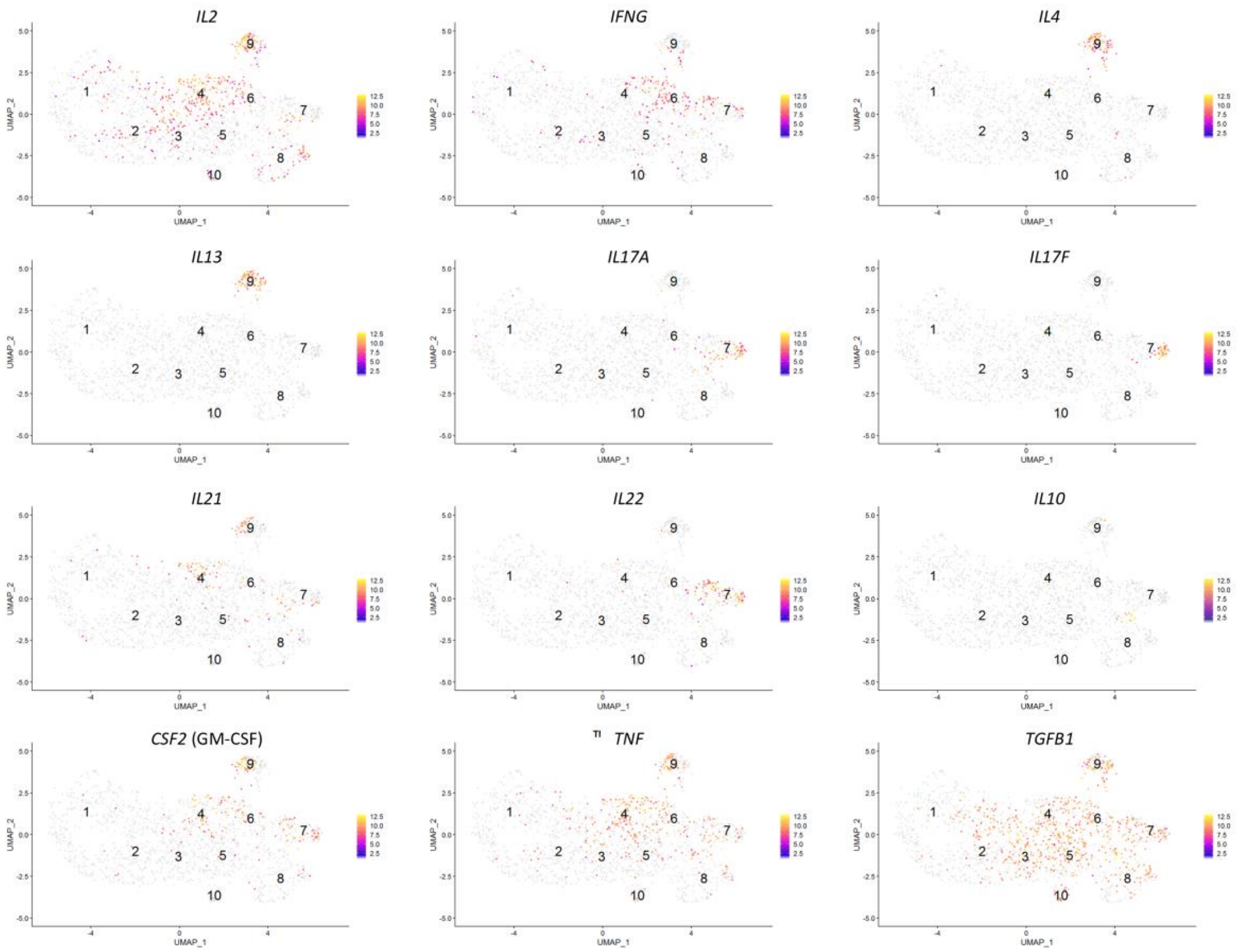
IL2
IFNG
IL4
IL13
IL17A
IL17F
IL21
IL22
IL10
CSF2 (GM-CSF)
TNF
TGFB1
UMAP_1
UMAP_2
12.5
10.0
7.5
5.0
2.5

b

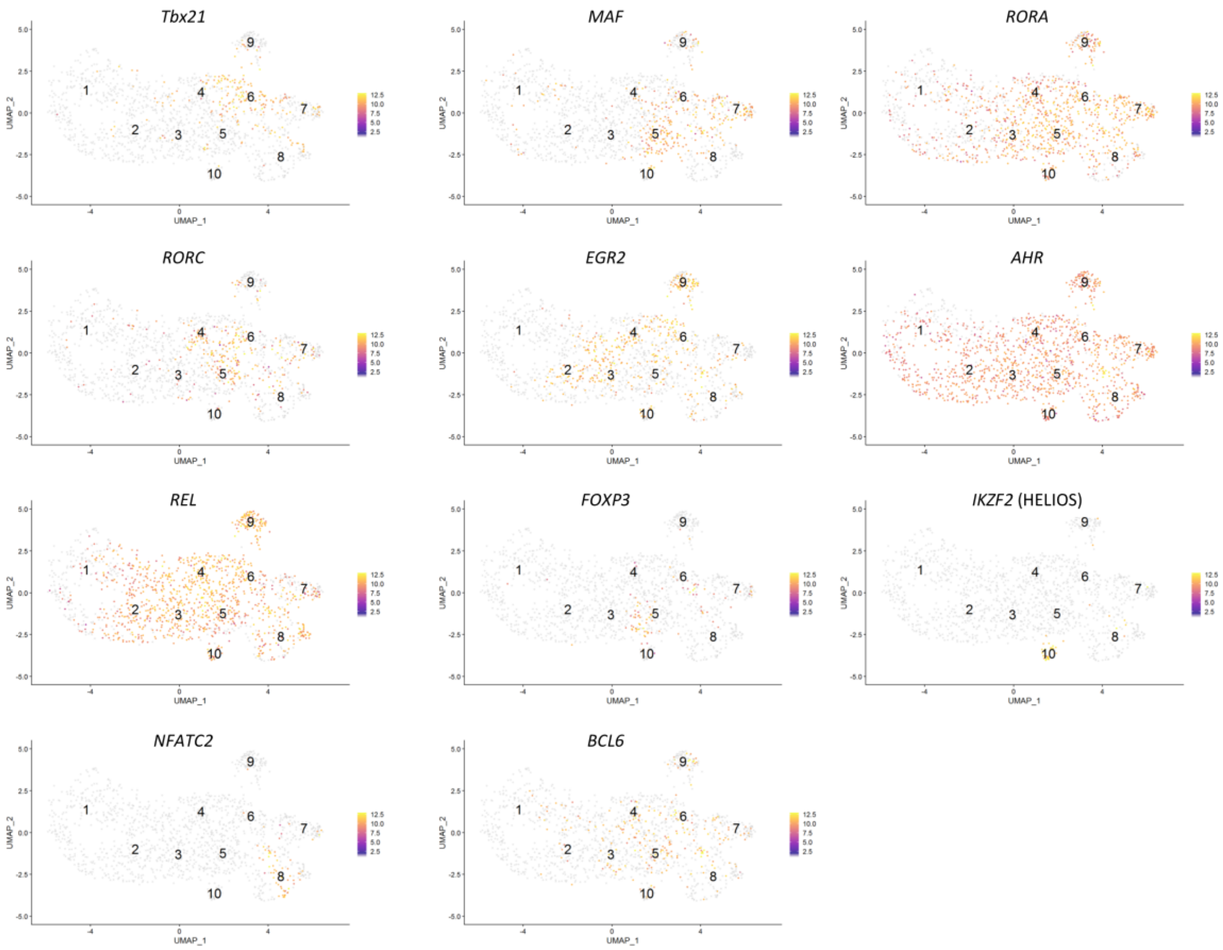
Tbx21
MAF
RORA
RORC
EGR2
AHR
REL
FOXP3
IKZF2 (HELIOS)
NFATC2
BCL6
UMAP_1
UMAP_2
1
2
3
4
5
6
7
8
9
10
12.5
10.0
7.5
5.0
2.5

c

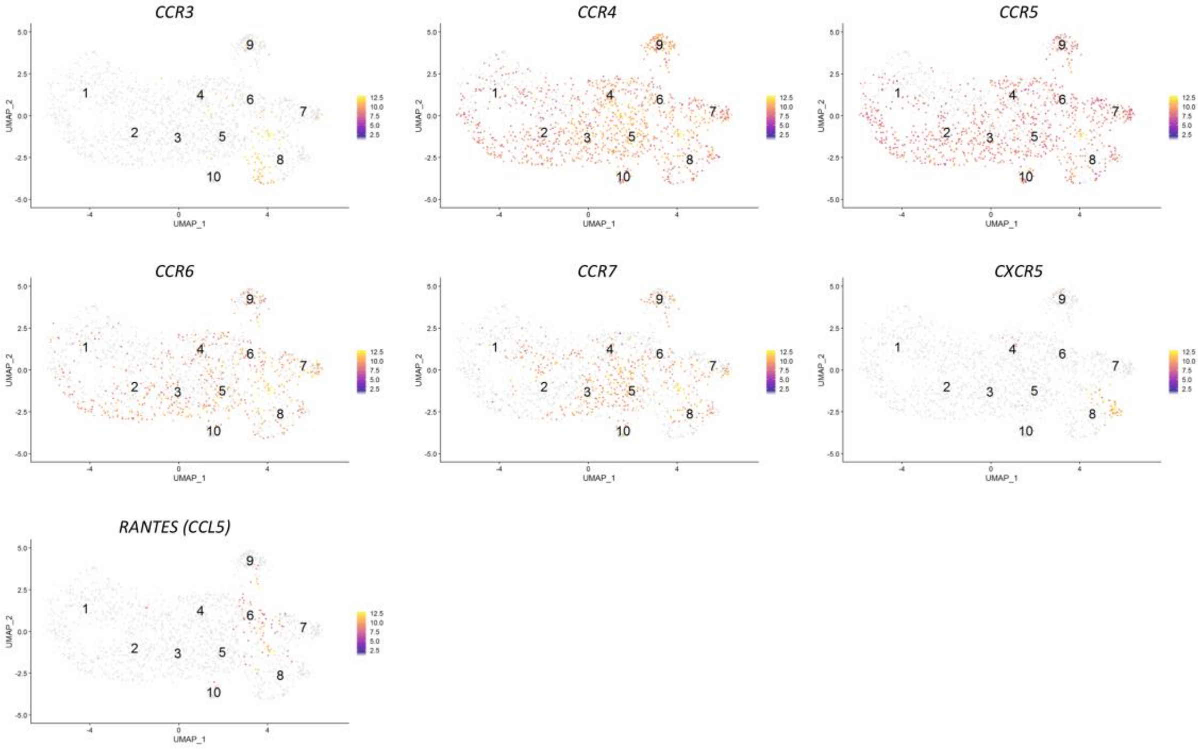
CCR3
CCR4
CCR5
CCR6
CCR7
CXCR5
RANTES (CCL5)
UMAP_1
UMAP_2
12.5
10.0
7.5
5.0
2.5

d

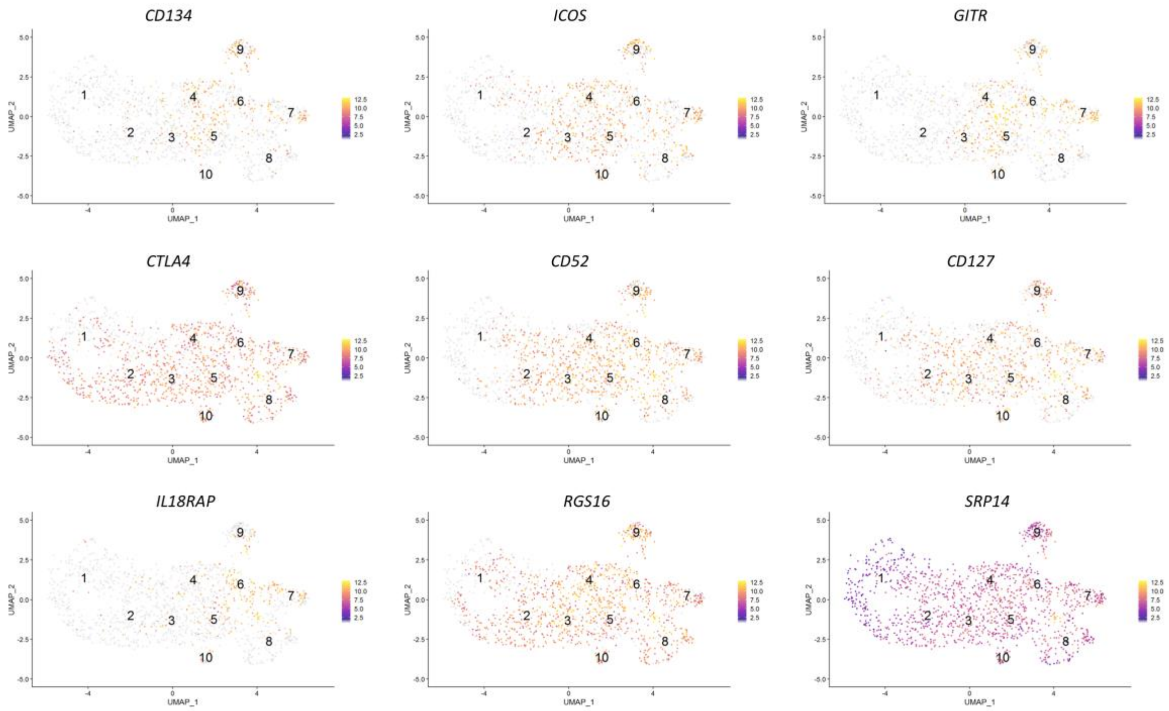

Supplementary Figure 8: Individual UMAP plots for cytokines (**A**), transcription factors (**B**), chemokines and chemokine receptors (**C**), and activation-related genes and other markers (**D**).

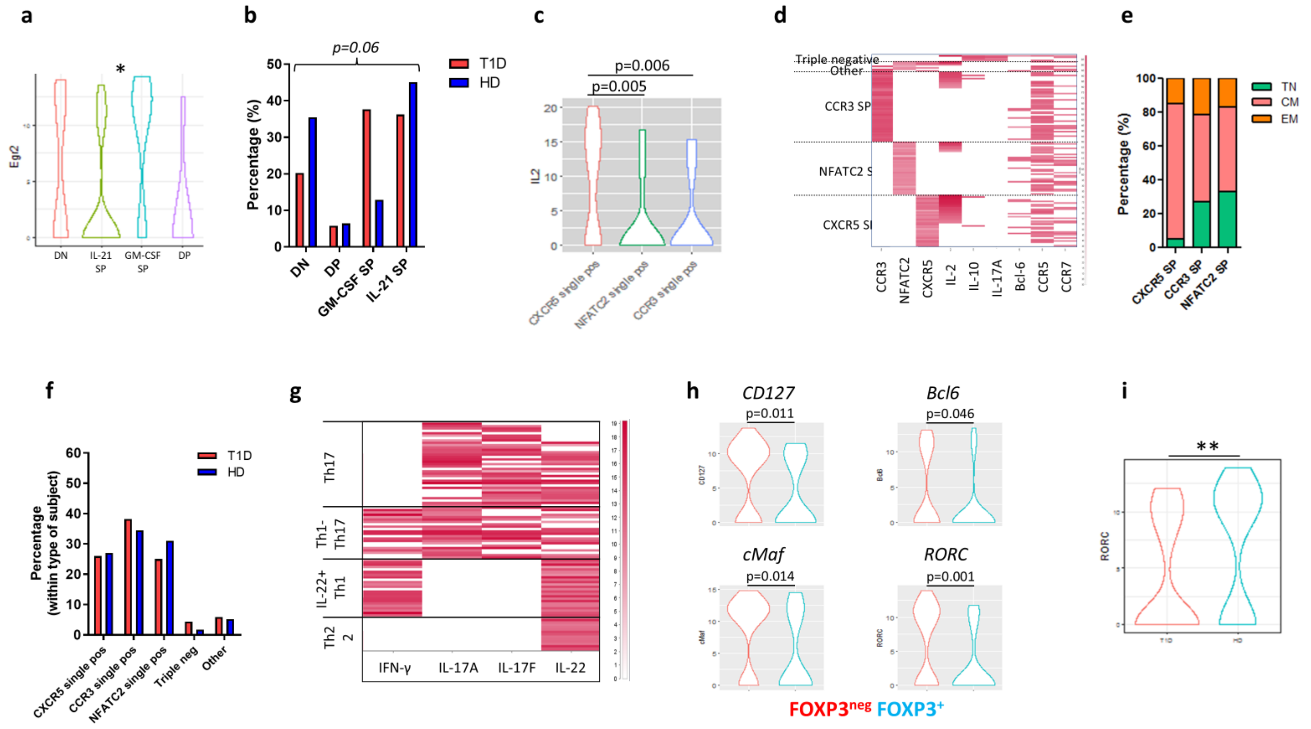

Supplementary Figure 9: **Features of specific gene expression clusters. A.** *Egr2* expression levels in the four different subclusters of cluster #4 (Kruskal-Wallis + Dunn. Bonferroni correction. p=0.0039). **B:** Subcluster distribution of cells from HD (white) and T1D patients (black) in the four different subclusters of cluster #4 (two sided Fisher's exact test. p=0.06). **C:** *IL-2* expression levels in cluster #8 subclusters (Kruskal-Wallis test, p=0.003, shown are post hoc Dunn p-values). **D:** Heatmap of selected relevant genes in cluster #8. **E:** Surface phenotype of three subclusters of cluster #8. **F:** Subcluster distribution of HD (white) and T1D (black) cells in Cluster #8. **G:** Heatmap of cytokines expressed by cluster #7 cells. **H:** Expression levels of selected genes in FOXP3^{neg} (red) and FOXP3^{+} (blue) cells from Cluster #5 (two-sided Mann-Whitney U test with Bonferroni correction). **I:** *RORC* expression levels in HD (blue) and T1D (red) cells of cluster #5 (two sided Mann Whitney U test with Bonferroni correction. p=0.007). *: p<0.05; **: p<0.01. ***: p<0.001.

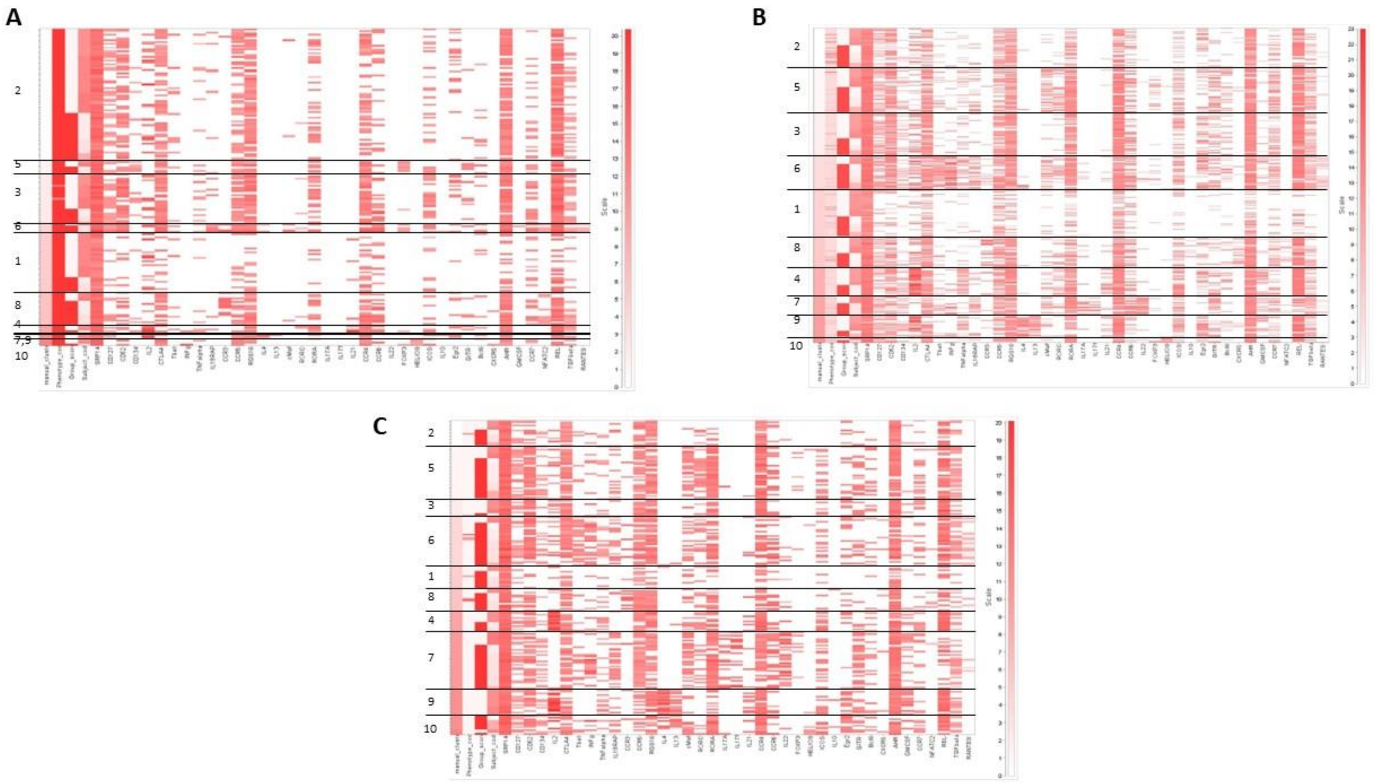

Supplementary Figure 10: Surface phenotype analysis. Heatmaps for cells with TN (**A**), CM (**B**) and EM (**C**) surface phenotypes. Cluster numbers are shown in the first columns. For the column "Group score", cells from HD are shown in red, and cells from T1D patients are shown in white.

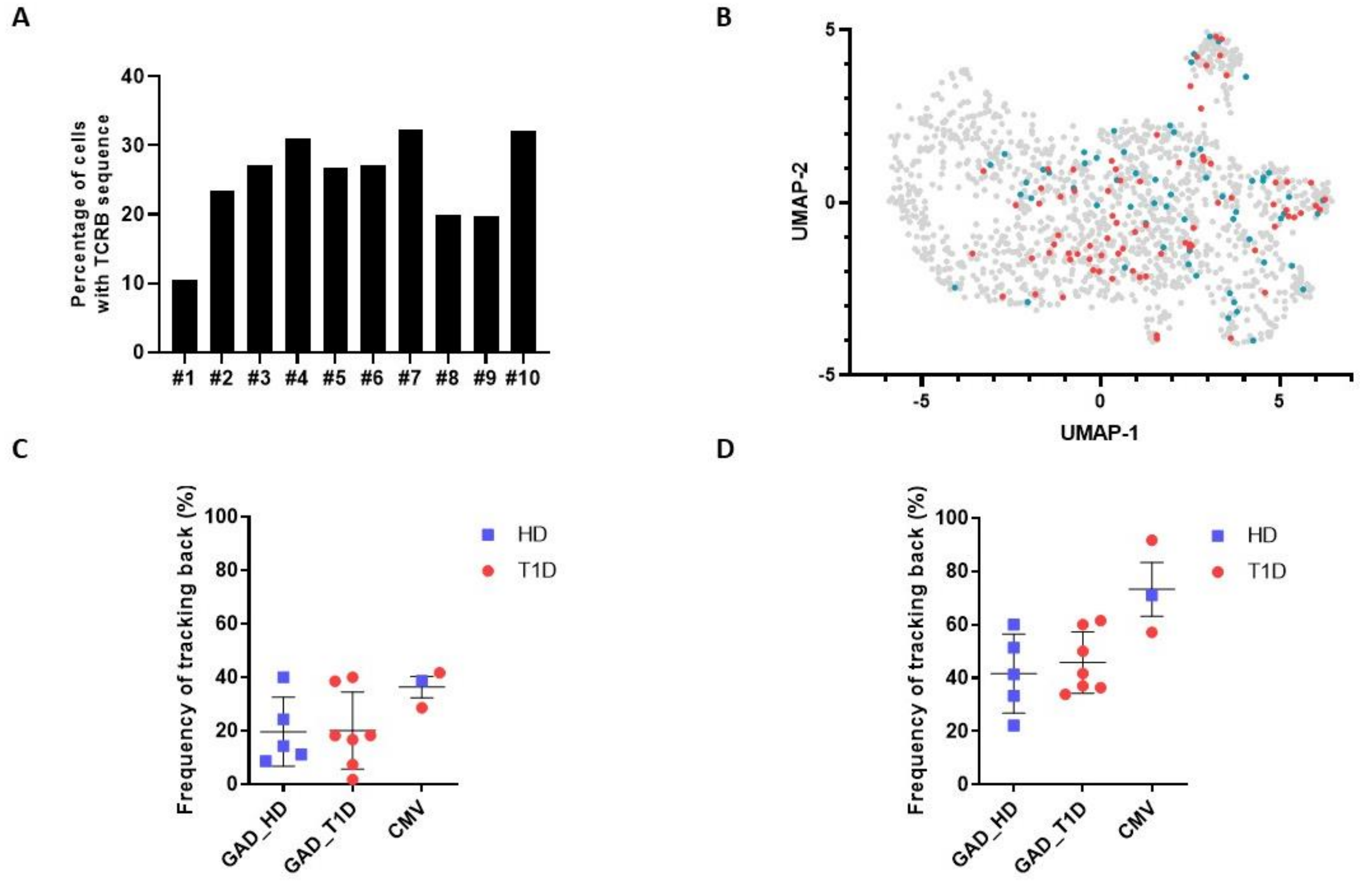

Supplementary Figure 11: A: Percentage of cells with TCRB CDR3 sequence per cluster. **B:** Distribution in the UMAP space of cells with known TCRA and TCRB. **C,D:** Frequencies of tracking back of TCRB CDR3 nucleotide **(C)** and amino acid **(D)** sequences (5 HD and 7 T1D patients for GAD65, and 1 HD and 2 T1D for CMV). Red: T1D. Blue: HD. Error bars represent standard deviations.

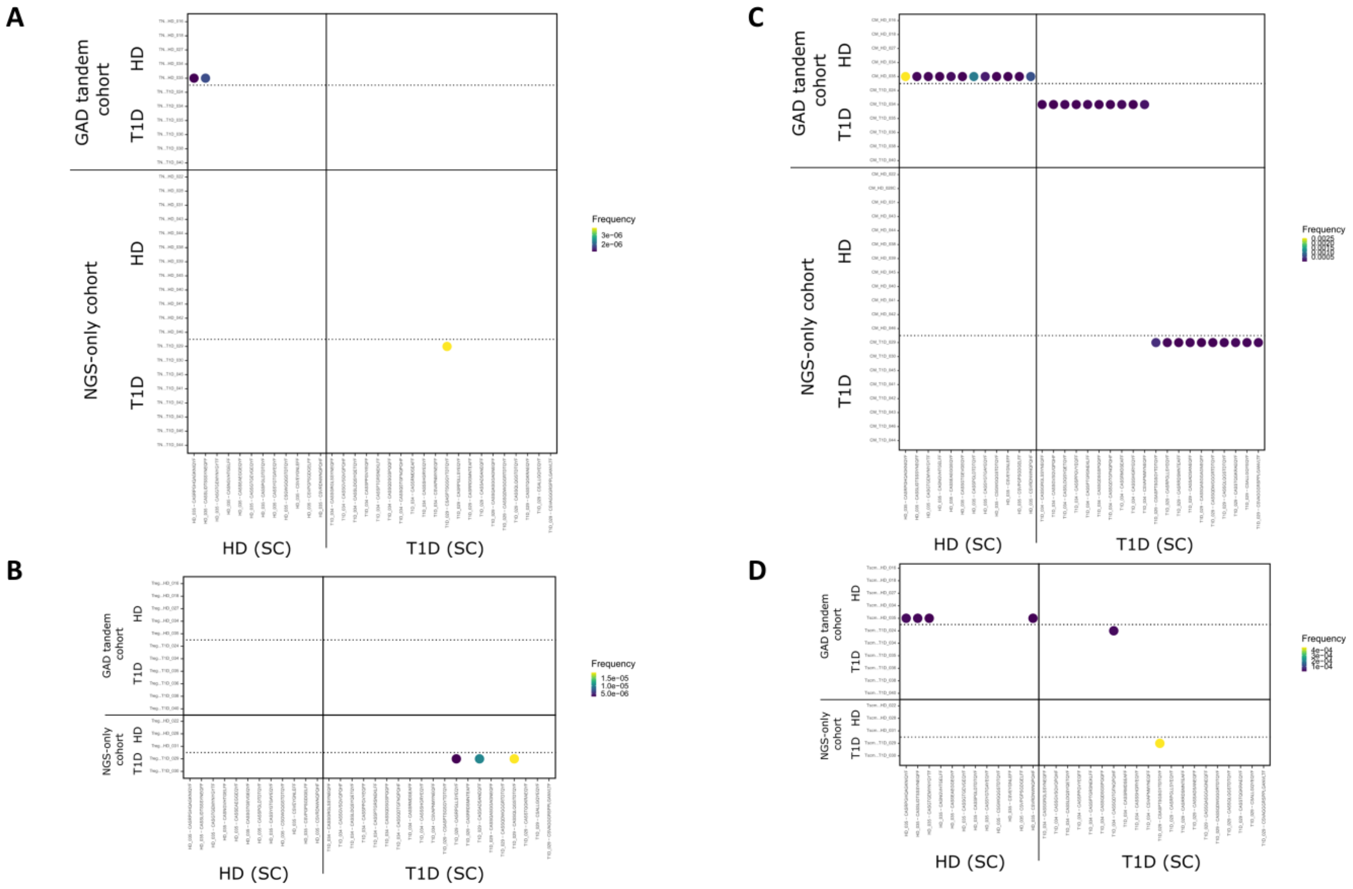

Supplementary Figure 12: CMV-specific TCRB CDR3 nucleotide sequences are found in peripheral immune cell subsets. A-D: We browsed the CMV-specific TCRB CDR3 nucleotide sequences into the 94 peripheral immune cell repertoires: 31 TN (**A**), 31 CM (**B**), 16 Treg (**C**) and 16 Tscm (**D**). We classified these repertoires into GAD65 tandem cohort (if both single-cell and deep sequencing took place) and NGS-only cohort (if we only performed deep sequencing). Colour represents frequency.

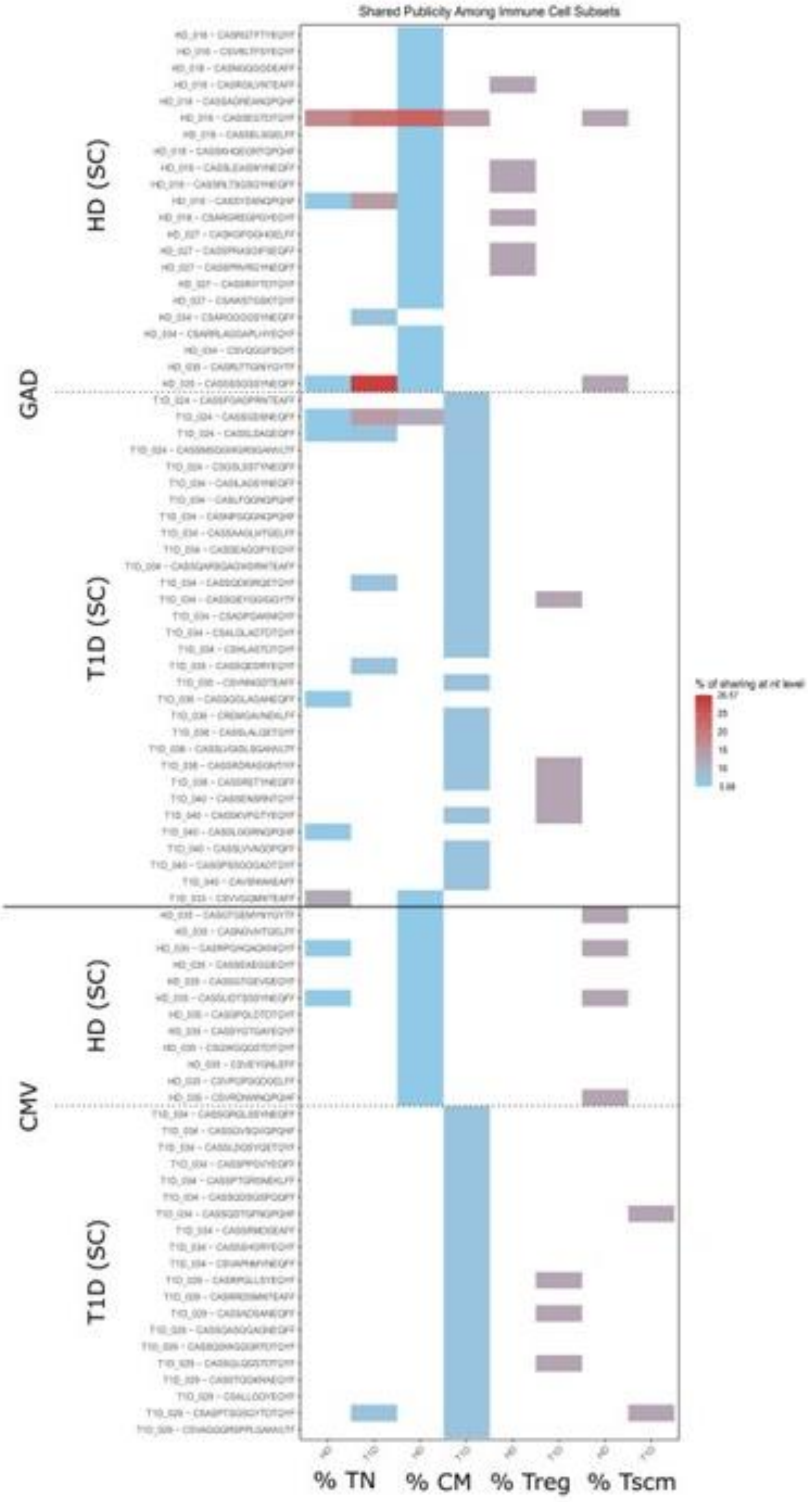

Supplementary Figure 13. Publicity of GAD65- and CMV-specific TCRB CDR3 nucleotide sequences among immune cell subsets. The heatmap shows the fraction of TN, CM, Treg and Tscm peripheral immune repertoires where each GAD65 and CMV nucleotide clonotype is found, showing that publicity seems to propagate from TN into CM, as some clonotypes public in TN cells are also public in CM cells.

a

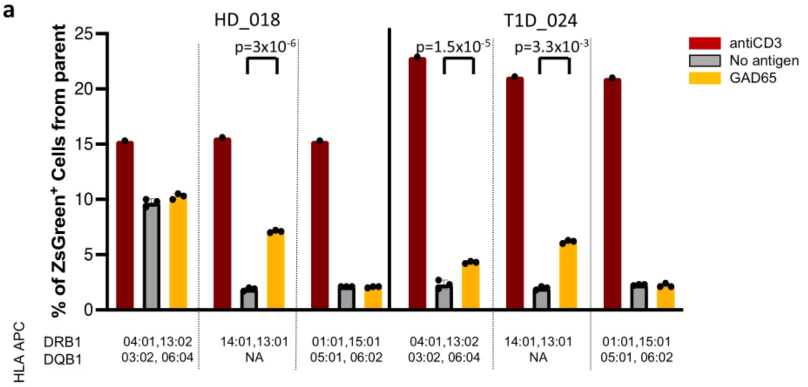

b

HLA	HD_018_#1_D04	T1D_024_#1_C03
DRB1	04:01, 14:01	04:05, 13:01
DQB1	03:02, 06:01	03:02, 06:03
CDR3A	CAGPGSGGSNYKLTF	CAVGAMEYGNKLVF
TRAV	25*01	8-3*
TRAJ	53*01	47*01
CDR3B	CASSYDSNQPQHF	CASSGDSNEQFF
TRBV	6-2*01	2*01
TRBJ	1-5*01	2-1*01

Supplementary Figure 14: Transduction of public GAD65 TCRs. Two selected public GAD65 TCR sequences were transduced into T-hybridoma cells and stimulated with whole GAD6565 antigen in the presence of HLA-matched antigen presenting cells (APCs, the live $CD3^-$ $CD4^-$ $CD8^-$ fraction of PBMCs). **A:** Y-axis shows the percentage of activated (ZsG^+) T cells after stimulation. Red bars show the results for the positive control (anti-CD3 antibody), grey bars for the negative control (no antigen) and yellow bars for GAD65 (two sided student t test corrected for multiple comparisons (Benjamini H)). No antigen and GAD6565 conditions were run in triplicates. **B:** HLA and TCR information of the two transduced TCRs. Activation profiles are consistent with presentation by DR or DQ on the HLA DRB1*1401 haplotype (HD_018) and the HLA DRB1*1301 haplotype (T1D_024). Mann-Whitney U test. ***: p<0.001. Error bars represent standard deviations.

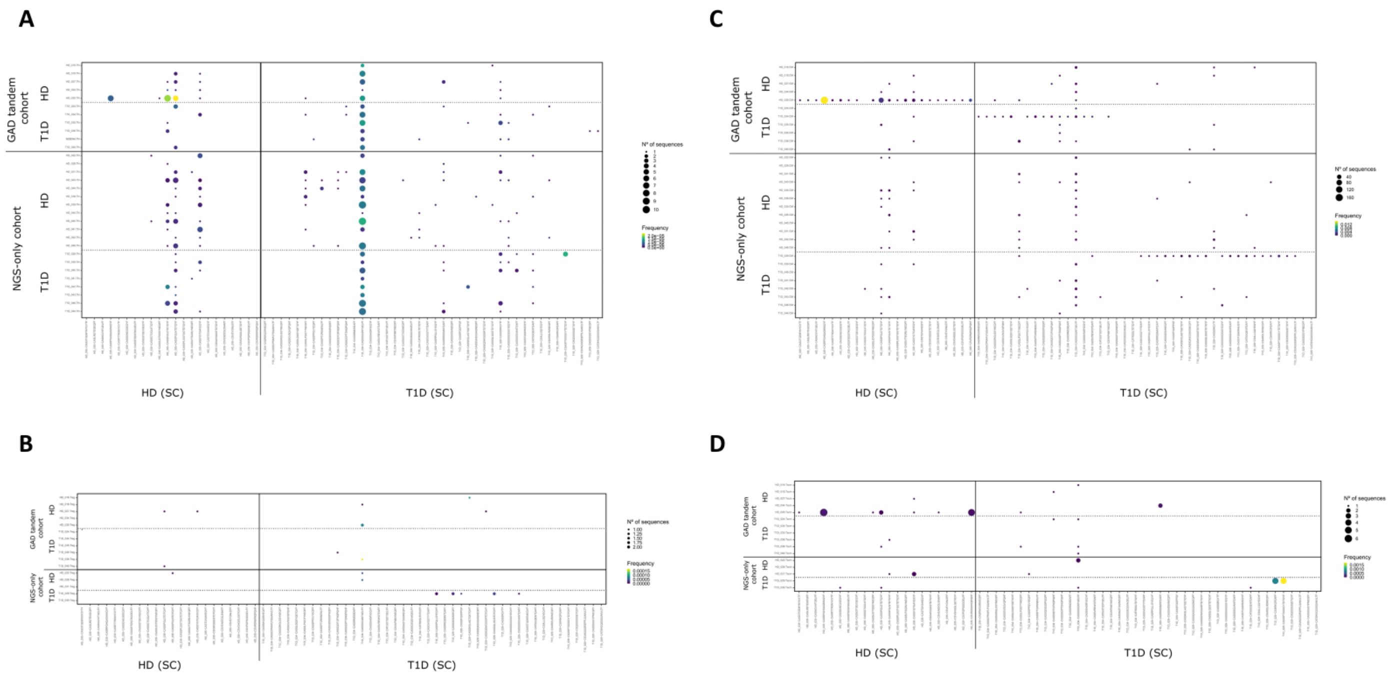

Supplementary Figure 15: CMV-specific amino acid clonotypes are public and convergent in peripheral immune cell subsets. A-D: We browsed the CMV-specific TCRB CDR3 amino acid clonotypes into the 94 peripheral immune cell repertoires: 31 TN (**A**), 31 CM (**B**), 16 Treg (**C**) and 16 Tscm (**D**). We classified these repertoires into GAD65 tandem cohort (if both single-cell and deep sequencing took place) and NGS-only cohort (if we only performed deep sequencing). Colour represents frequency, while dot size represents numbers of unique TCRB CDR3 nucleotide sequences coding for a given amino acid clonotype.

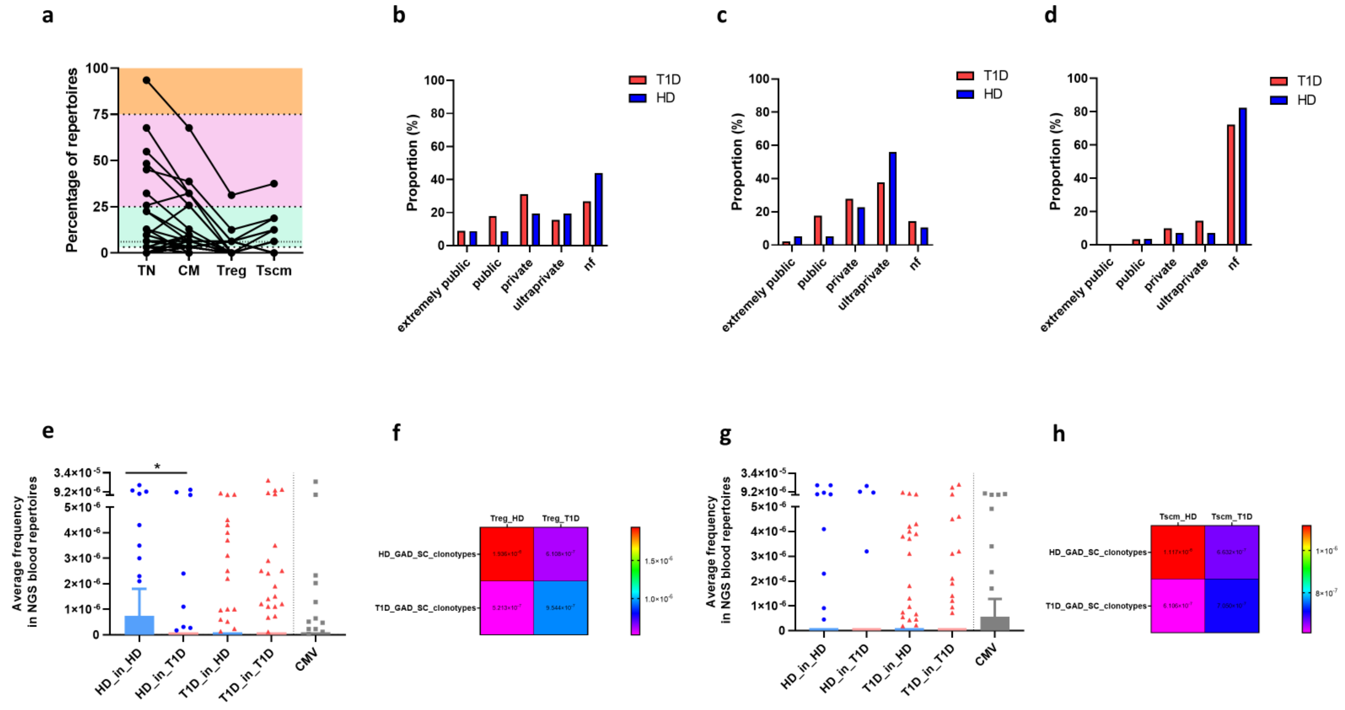

Supplementary Figure 16: A: Percentage of peripheral immune repertoires where each CMV-specific TCRB CDR3 amino acid clonotype is tracked into. Orange shade: extremely public clonotypes (present in ≥75% of individuals). Pink shade: public clonotypes (present in 25.0%-74.9% of individuals). Green shade: private clonotypes (present in 3.23%-24.9% of individuals) or ultraprivate (only found in the same single-cell donor, 3.23%). For Treg and Tscm, the threshold for "ultraprivate" is 5.88 and 6.25 respectively (faint dotted lines). **B-D:** Proportions of each publicity-based GAD65 clonotype category in TN (**B**), CM (**C**) and Tscm (**D**) repertoires (two sided Chi-square test, p=0.13, p= 0.07 and p=0.48, respectively). White: HD. Black: T1D). **E-H:** Frequency of GAD65-specific TCRB CDR3 amino acid clonoytpes in Treg (**E,F**) and Tscm (**G,H**) peripheral immune repertoires, disaggregated by type of donor (57 clonotypes for HD, 90 for T1D and 64 for CMV).. **E,G** shows Tukey boxplots: boxes are interquartile range (25% to 75%), whisker (up) is 75th percentile plus 1.5 times the interquartile range (IQR). The centre is the 50th percentile. Individual dots are values that are greater than the whisker. Two sided Mann Whitney U test with Bonferroni correction (*: p=0.026). The frequency of each clonotype was calculated as the average frequency for all nucleotide sequences coding for the same amino acid sequence (See Methods section). **F,H:** heatmaps for mean values of data shown in **E** and **G** (two sided Mann Whitney U test with Bonferroni correction**)**.

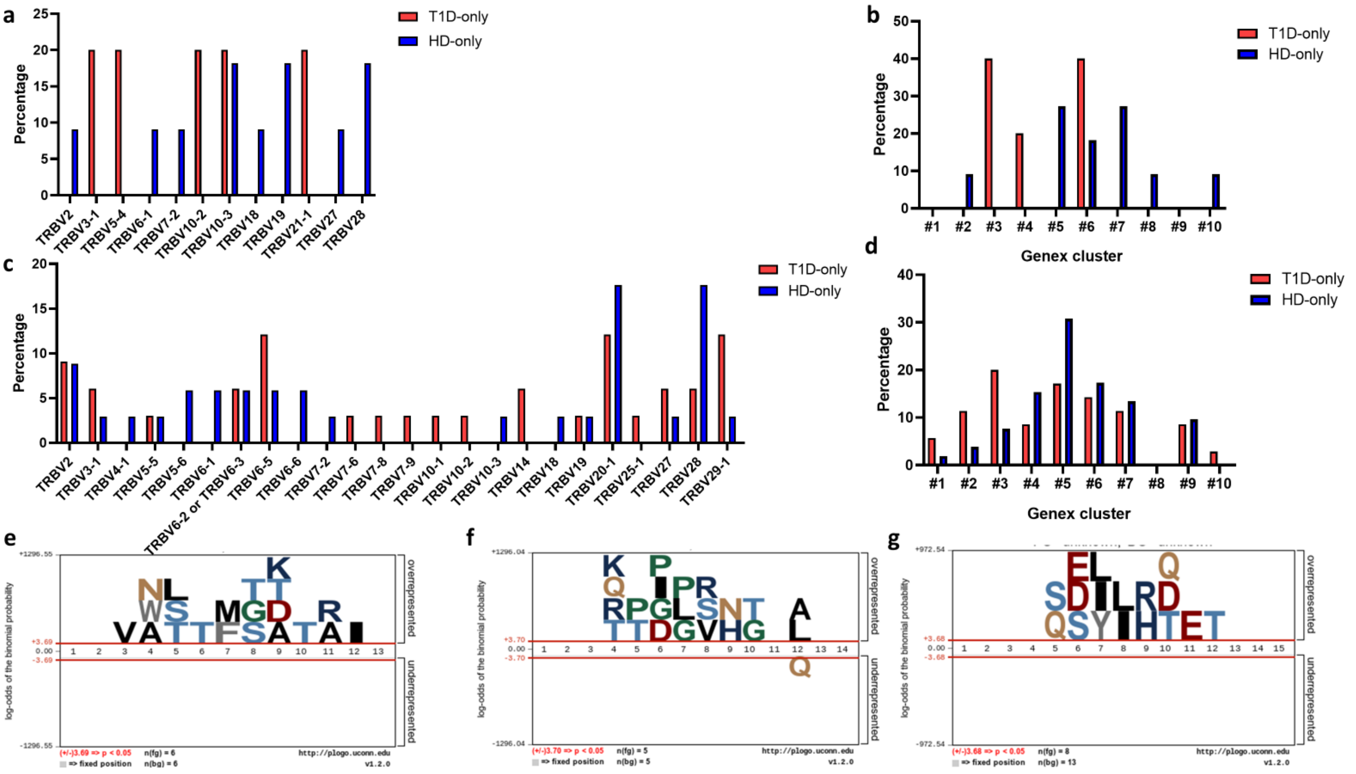

Supplementary Figure 17: V-gene usage, genex cluster distribution and amino acid usage of HD-only and T1D-only GAD65 clonotypes. We identified GAD65 clonotypes derived from HD and appearing in peripheral repertoires from HD but not from T1D patients (“HD-only”), and GAD65 clonotypes derived from T1D patients and appearing in peripheral repertoires from T1D patients but not from HD (“T1D-only”) and analyzed their features. **A,B:** V-gene usage (**A**) and genex cluster distribution (**B**) of GAD65 clonotypes found in TN peripheral repertoires (white: HD-only clonotypes. Black: T1D-only clonotypes). **C,D:** V-gene usage (**C**) and genex cluster distribution (**D**) of GAD65 clonotypes found in CM peripheral repertoires (white: HD-only clonotypes. Black: T1D-only clonotypes). **E-G:** preferential amino acid usage of T1D-only *versus* HD-only GAD65 clonotypes found in CM peripheral repertoires. Logos were separately generated for CDR3B sequences of 13 (**E**), 14 (**E**) and 15 (**E**) amino acids. Residue heights are scaled relative to their statistical significance, with residues stacked from most to least significant.

Supplementary tables

Supplementary Table 1: demographics.

	n	Age (years. Mean ± SD)	Number of females	Months since diagnosis (mean ± SD)	Number of DR3+ donors	Number of DR4+ donors	GAD65A Units (mean ± SD)	IA2 Units (mean ± SD)	ZnT8R Units (mean ± SD)	ZnT8W Units (mean ± SD)
ELISPOT										
HD	36	31.9 ± 7.0	21/36	N/A	11/36	8/36	0.45 ± 1.56	0.01 ± 0.03	0.14 ± 0.03	0.15 ± 0.05
T1D	40	28.9 ± 7.2	13/36	3.2 ± 2.4	15/40	13/40	439.22 ± 414.63	93.95 ± 159.79	14.52 ± 26.46	25.09 ± 36.35
AIM assay										
HD	10	29.4 ± 6.4	6/10	N/A	4/10	4/10	1.56 ± 3.19	0.01 ± 0.03	0.14 ± 0.02	0.16 ± 0.06
T1D	11	30.2 ± 7.6	6/11	3.8 ± 2.1	5/11	6/11	540.38 ±434.91	140.62 ± 196.52	23.72 ± 33.61	42.12 ± 41.66
Single cell gene expression										
HD	5	31.4 ± 8.8	2/5	N/A	1/5	4/5	0.00 ± 0.00	0.02 ± 0.04	0.13 ± 0.00	0.18 ± 0.07
T1D	7	28.3 ± 5.9	2/7	3.3 ± 1.8	3/7	6/7	499.60 ± 445.70	216.48 ± 214.10	31.59 ± 36.37	56.11 ± 37.79
Deep sequencing										
HD	17	28.6 ± 5.8	9/17	N/A	6/17	5/17	0.00 ± 0.00	0.01 ± 0.03	0.13 ± 0.02	0.16 ± 0.06
T1D	14	30.3 ± 7.7	4/14	5.3 ± 3.5	5/14	8/14	589.71 ±405.16	136.33 ± 195.56	47.28 ±34.18	35.94 ±20.34

N/A: not analyzed. Positive threshold for autoantibodies: GAD65A (33 units), IA-2A (1.4 units), ZnT8R&W (1.8 units).

Supplementary Table 2: Statistics of AIM assay

Donor	% GAD65 CD154+ CD69+ cells	% GAD65 diluent CD154+ CD69+ cells	Delta	Delta rank	Number of sorted GAD65 CD154+ CD69+ cells
HD_035	0.0180	0.0029	0.0151	1	123
T1D_034	0.0160	0.0017	0.0143	2	282
HD_027	0.0150	0.0026	0.0124	3	158
T1D_039	0.0160	0.0065	0.0095	4	N/A
T1D_038	0.0160	0.0072	0.0089	5	168
T1D_033	0.0099	0.0016	0.0082	6	151
HD_034	0.0130	0.0048	0.0082	7	103
T1D_030	0.0120	0.0042	0.0078	8	N/A
HD_002	0.0100	0.0024	0.0076	9	N/A
T1D_029	0.0090	0.0033	0.0058	10	N/A
T1D_035	0.0074	0.0019	0.0055	11	150
HD_016	0.0063	0.0016	0.0047	12	63
T1D_024	0.0083	0.0037	0.0046	13	135
HD_018	0.0071	0.0032	0.0039	14	144
HD_031	0.0071	0.0032	0.0039	15	N/A
T1D_037	0.0075	0.0041	0.0034	16	N/A
HD_037	0.0043	0.0023	0.0020	17	N/A
T1D_040	0.0029	0.0014	0.0016	18	154
T1D_036	0.0024	0.0009	0.0015	19	55
HD_022	0.0032	0.0022	0.0011	20	N/A
HD_028	0.0020	0.0010	0.0010	21	N/A

Shown are frequencies of GAD65 and GAD65 diluent $CD154^+$ $CD69^+$ cells, delta values (percentage GAD65 $CD154^+$ $CD69^+$ cells minus percentage GAD65 diluent $CD154^+$ $CD69^+$ cells), rank of the samples based upon delta values, and number of sorted single cells. The table is sorted from highest to lowest delta values.

Supplementary Table 3: GAD65 TCRB CDR3 clonotypes sharing similar features among different donors.

Cluster name	Number of donors	Number of unique TCR CDR3B's	Number of cells	V-gene enrichment (p-value)	Region significantly enriched (p-value)
**CRG-CASSLPTEYKQYF**	8/12 (4 HD, 4 T1D)	18	21	No (p=0.07)	YK, SYK (p=0.001)
**CRG-CASSYQWVGSGANVLTF**	2 (both T1D)	5	7	Yes (p=0.001)	SYQ (p=0.001)
**CRG-CASQTSGRYNEQFF**	2 (1HD, 1 T1D)	5	6	Yes (p=0.001)	GRYN, SGRY and NKS (p=0.001)
**CRG-CATQGPGGGQKTQYF**	3/12 (2HD, 1 T1D)	4	5	No	QKT (p=0.001)

GLIPH algorithm was applied to GAD65-specific clonotypes, and convergence groups consisting of cells appearing in different donors are shown. Nine other groups consisted of two clonotypes appearing in two cells from the same donor. V-gene and region enrichment was analyzed comparing the observed values to the stimulated ones using GLIPH (one sided, reference 62).

Supplementary Table 4: tracking back statistics at the nucleotide (nt) and amino acid (aa) levels.

Donor	GAD65					CMV						
	Number of productive unique TCRB CDR3	Number of tracked back clonoytpes (nt level)	Frequency of tracking back (nt level, percentage)	Number of tracked back clonoytpes (aa level)	Frequency of tracking back (aa level, percentage)	Number of productive unique TCRB CDR3	Number of tracked back clonoytpes (nt level)	Frequency of tracking back (nt level, percentage)	Number of tracked back clonoytpes (aa level)	Frequency of tracking back (aa level, percentage)	CMV IgG	CMV IgM
HD_016	18	2	11.1	4	22.2	*n/a*	*n/a*	*n/a*	*n/a*	*n/a*	*n/a*	*n/a*
HD_018	37	9	24.3	19	51.4	*n/a*	*n/a*	*n/a*	*n/a*	*n/a*	*n/a*	*n/a*
HD_027	58	5	8.6	24	41.4	*n/a*	*n/a*	*n/a*	*n/a*	*n/a*	*n/a*	*n/a*
HD_034	21	3	14.3	7	33.3	*n/a*	*n/a*	*n/a*	*n/a*	*n/a*	*n/a*	*n/a*
HD_035	5	2	40.0	3	60.0	31	12	38.7	22	71.0	POS	Neg
T1D_029	*n/a*	*n/a*	*n/a*	*n/a*	*n/a*	24	10	41.7	22	91.7	POS	POS
T1D_024	13	5	38.5	8	61.5	*n/a*	*n/a*	*n/a*	*n/a*	*n/a*	*n/a*	*n/a*
T1D_033	56	1	1.8	19	33.9	*n/a*	*n/a*	*n/a*	*n/a*	*n/a*	*n/a*	*n/a*
T1D_034	60	11	18.3	30	50.0	35	10	28.6	20	57.1	Neg	Neg
T1D_035	27	2	7.4	10	37.0	*n/a*	*n/a*	*n/a*	*n/a*	*n/a*	*n/a*	*n/a*
T1D_036	11	2	18.2	4	36.4	*n/a*	*n/a*	*n/a*	*n/a*	*n/a*	*n/a*	*n/a*
T1D_038	24	4	16.7	10	41.7	*n/a*	*n/a*	*n/a*	*n/a*	*n/a*	*n/a*	*n/a*
T1D_040	15	6	40.0	9	60.0	*n/a*	*n/a*	*n/a*	*n/a*	*n/a*	*n/a*	*n/a*

n/a: not analyzed.

Supplementary Table 5: antibodies and dilutions used.

Target	Clone	Catalog Number	Manufacturer	Dilution
CD14	TuK4	MHCD1428	Invitrogen	1/50
CD19	SJ25-C1	MHCD1928	Invitrogen	1/50
CD3	SK7	641415	BD Biosciences	1/50
CD154	TRAP1	555700	BD Biosciences	1/40
CD69	FN50	555530	BD Biosciences	1/38
CD4	SK3	46-0047-42	eBiosciences	1/33
CD45RO	UCHL1	337168	BD Biosciences	1/42
CD27	O323	302830	Biolegend	1/83
CD95	DX2	561978	BD Biosciences	1/33
