# Supplementary material for: Physiological and pathogenic T cell autoreactivity converge in type 1 diabetes
Source: Nat Commun. 2024 Oct 29;15:9204. doi: 10.1038/s41467-024-53255-9 (PMC11522472; doi:10.1038/s41467-024-53255-9)
Supplement: Supplementary file 3 — Reporting Summary [file 41467_2024_53255_MOESM3_ESM.pdf]

nature portfolio

Corresponding author(s): Iria Gomez-Tourino

Last updated by author(s): Sep 16, 2024

Reporting Summary

Nature Portfolio wishes to improve the reproducibility of the work that we publish. This form provides structure for consistency and transparency in reporting. For further information on Nature Portfolio policies, see our Editorial Policies and the Editorial Policy Checklist.

Statistics

For all statistical analyses, confirm that the following items are present in the figure legend, table legend, main text, or Methods section.

n/a	Confirmed	
☐	☒	The exact sample size (*n*) for each experimental group/condition, given as a discrete number and unit of measurement
☐	☒	A statement on whether measurements were taken from distinct samples or whether the same sample was measured repeatedly
☐	☒	The statistical test(s) used AND whether they are one- or two-sided<br>*Only common tests should be described solely by name; describe more complex techniques in the Methods section.*
☐	☒	A description of all covariates tested
☐	☒	A description of any assumptions or corrections, such as tests of normality and adjustment for multiple comparisons
☐	☒	A full description of the statistical parameters including central tendency (e.g. means) or other basic estimates (e.g. regression coefficient) AND variation (e.g. standard deviation) or associated estimates of uncertainty (e.g. confidence intervals)
☐	☒	For null hypothesis testing, the test statistic (e.g. *F*, *t*, *r*) with confidence intervals, effect sizes, degrees of freedom and *P* value noted<br>*Give P values as exact values whenever suitable.*
☒	☐	For Bayesian analysis, information on the choice of priors and Markov chain Monte Carlo settings
☒	☐	For hierarchical and complex designs, identification of the appropriate level for tests and full reporting of outcomes
☐	☒	Estimates of effect sizes (e.g. Cohen's *d*, Pearson's *r*), indicating how they were calculated

Our web collection on statistics for biologists contains articles on many of the points above.

Software and code

Policy information about availability of computer code

Data collection	No specific software was used to collect the data
Data analysis	SPSS (IBM), GraphPad Prism 8, KNIME 4.5.1, Seurat package version 3.1.3, MAST package version 1.12 and GLIPH version 1.0

For manuscripts utilizing custom algorithms or software that are central to the research but not yet described in published literature, software must be made available to editors and reviewers. We strongly encourage code deposition in a community repository (e.g. GitHub). See the Nature Portfolio guidelines for submitting code & software for further information.

Data

Policy information about availability of data

All manuscripts must include a data availability statement. This statement should provide the following information, where applicable:

- Accession codes, unique identifiers, or web links for publicly available datasets
- A description of any restrictions on data availability
- For clinical datasets or third party data, please ensure that the statement adheres to our policy

TCRB CDR3 sequencing data of peripheral immune cell subsets have been deposited and made public in the Open Science Framework database (DOI 10.17605/OSF.IO/YDGTV)70: http://osf.io/ydgtv/), in GEO (GSE272431), and published in reference41. GAD- and CMV-specific clonotype sequences have been deposited in GenBank (IDs PP952812-PP953496). The TCRB CDR3 sequencing data, along with associated study metadata, for both the peripheral immune cell subsets and the GAD- and CMV-specific clonotypes are stored in the AIRR Data Commons and can be searched and downloaded using the iReceptor Gateway86, 87 (https://

gateway.ireceptor.org) study IDs "DOI:10.21417/B7C88S" and "IR-T1D-000003" respectively. Source data are provided with this paper. All other data are available in the Supplementary Data files.

Research involving human participants, their data, or biological material

Policy information about studies with human participants or human data. See also policy information about sex, gender (identity/presentation), and sexual orientation and race, ethnicity and racism.

Reporting on sex and gender	Findings apply to both sexes, and sex was considered in the study design to match the healthy donor and patient cohorts, so they included equal numbers of male and female participants. Gender information was not collected. Sex-based analysis was not performed due to low sample size to enable meaningful conclusions.
Reporting on race, ethnicity, or other socially relevant groupings	Race, ethnicity, or other socially relevant grouping information was not collected in this study.
Population characteristics	The study includes two cohorts, one consisting of healthy donors (defined as not having any infectious or autoimmune disease at the time of blood draw), and one consisting of patients with Type 1 Diabetes. Population characteristics are detailed in Supplementary Table 1.
Recruitment	Type 1 Diabetes patients were recruited during their visits to the hospital. Healthy donors were volunteers of similar age and sex. Further information in the Methods section. There were no self-selection biases
Ethics oversight	The study was approved by the National Research Ethics Service, NRES Committee London-Bromley, REC reference 08/H0805/14. All uses of human material have been approved, and all recruited volunteers provided written informed consent. Patients were compensated for their time.

Note that full information on the approval of the study protocol must also be provided in the manuscript.

Field-specific reporting

Please select the one below that is the best fit for your research. If you are not sure, read the appropriate sections before making your selection.

☒ Life sciences ☐ Behavioural & social sciences ☐ Ecological, evolutionary & environmental sciences

For a reference copy of the document with all sections, see nature.com/documents/nr-reporting-summary-flat.pdf

Life sciences study design

All studies must disclose on these points even when the disclosure is negative.

Sample size	Sample size calculations were not performed prior to the development of the study. Sample sizes were chosen based upon sample sizes used previously from us and others, which showed statistical power.
Data exclusions	No data were excluded from the analysis
Replication	High sample sizes and depth of T cell receptor sequencing were used as measures to confirm replication. Reproducibility of the experiments was confirmed either by conducting experimental replicates, by including intra and inter-experiment controls, or by analyzing several donors. We took as potential covariates age, sex and DR3 and DR4 haplotypes. We controlled for them by including donors with comparable ages, and sex/DR3/DR4 frequencies in the two groups to compare (HD and T1D patients) for each experimental setting.
Randomization	There were no randomization groups in our study.
Blinding	Investigators were not blinded, as disease status was the main variable to analyze.

Reporting for specific materials, systems and methods

We require information from authors about some types of materials, experimental systems and methods used in many studies. Here, indicate whether each material, system or method listed is relevant to your study. If you are not sure if a list item applies to your research, read the appropriate section before selecting a response.

Materials & experimental systems

n/a	Involved in the study
☐	☒ Antibodies
☐	☒ Eukaryotic cell lines
☒	☐ Palaeontology and archaeology
☒	☐ Animals and other organisms
☒	☐ Clinical data
☒	☐ Dual use research of concern
☒	☐ Plants

Methods

n/a	Involved in the study
☒	☐ ChIP-seq
☐	☒ Flow cytometry
☒	☐ MRI-based neuroimaging

Antibodies

Antibodies used	(indicated in brackets is clone name, catalog number and volume in µL used to stain 2 × 106 PBMCs): anti-CD14 (TuK4, MHCD1428, 2 µL), anti- CD19 (SJ25-C1, MHCD1928, 2 µL) (Invitrogen); anti-CD3 (SK7, 641415, 2 µL), anti-CD154 (TRAP1, 555700, 2.5 µL), anti-CD69 (FN50, 555530, 2.5 µL), anti-CD45-RO (UCHL1, 337168, 2.4 µL), anti-CD95 (DX2, 561978, 3 µL) (all from BD Biosciences); anti-CD4 (SK3, 46-0047-42, 3 µL. eBiosciences), anti-CD27 (O323, 302830, 1.2 µL. Biolegend; and LIVE/DEAD Fixable ViVid Dead Cell Stain (Molecular Probes, L34955).
Validation	All antibodies are primary antibodies, validated by the manufacturers described above (Invitrogen, BD Biosciences, eBiosciences, Biolegend, Molecular Probes) for their use in flow cytometry.

Eukaryotic cell lines

Policy information about cell lines and Sex and Gender in Research

Cell line source(s)	5KC hybridoma cell line: https://pubmed.ncbi.nlm.nih.gov/33732772/ Professor Maki Nakayama, Barbara Davis Center for Childhood Diabetes, University of Colorado School of Medicine, Aurora, CO, United States
Authentication	Cells were not authenticated by us, but by the collaborator providing them
Mycoplasma contamination	The cell line was not tested for Mycoplasma contamination
Commonly misidentified lines (See ICLAC register)	No misidentified lines

Plants

Seed stocks	*Report on the source of all seed stocks or other plant material used. If applicable, state the seed stock centre and catalogue number. If plant specimens were collected from the field, describe the collection location, date and sampling procedures.*
Novel plant genotypes	*Describe the methods by which all novel plant genotypes were produced. This includes those generated by transgenic approaches, gene editing, chemical/radiation-based mutagenesis and hybridization. For transgenic lines, describe the transformation method, the number of independent lines analyzed and the generation upon which experiments were performed. For gene-edited lines, describe the editor used, the endogenous sequence targeted for editing, the targeting guide RNA sequence (if applicable) and how the editor was applied.*
Authentication	*Describe any authentication procedures for each seed stock used or novel genotype generated. Describe any experiments used to assess the effect of a mutation and, where applicable, how potential secondary effects (e.g. second site T-DNA insertions, mosiacism, off-target gene editing) were examined.*

Flow Cytometry

Plots

Confirm that:

☒ The axis labels state the marker and fluorochrome used (e.g. CD4-FITC).

☒ The axis scales are clearly visible. Include numbers along axes only for bottom left plot of group (a 'group' is an analysis of identical markers).

☒ All plots are contour plots with outliers or pseudocolor plots.

☒ A numerical value for number of cells or percentage (with statistics) is provided.

Methodology

Sample preparation	PBMCs were immediately isolated from heparinised blood by density gradient centrifugation using Lymphoprep (Axis-Shield PoC AS, Oslo, Norway) as indicated by the manufacturer, and used always fresh. For TCR transduction experiments, cell

	cocultures were stained with Draq7TM (BioStatus).
Instrument	BD FACSAria II, BD FACSCanto II or LSRFortessa
Software	FACSDiva and FlowJo (BD)
Cell population abundance	Abundance of post-sort populations is shown in Figure 1, Supplementary Figures 2, 5 and 7, Table 1 and reference 41
Gating strategy	The gating and sorting strategy is shown in Supplementary Figure 3

☒ Tick this box to confirm that a figure exemplifying the gating strategy is provided in the Supplementary Information.
