# Supplementary material for: Physiological and pathogenic T cell autoreactivity converge in type 1 diabetes
Source: Nat Commun. 2024 Oct 29;15:9204. doi: 10.1038/s41467-024-53255-9 (PMC11522472; doi:10.1038/s41467-024-53255-9)
Supplement: Supplementary file 4 — Description of Additional Supplementary Files [file 41467_2024_53255_MOESM4_ESM.pdf]

Description of Additional Supplementary Files

File Name: Supplementary Data 1

Description: Experiments ran for each donor.

File Name: Supplementary Data 2

Description: Genes defining each gene expression cluster.

File Name: Supplementary Data 3

Description: GAD clonotypes tracked back into TN peripheral repertoires and found only in HD (HD-only) or only in T1D patients (T1D-only).

File Name: Supplementary Data 4

Description: GAD clonotypes tracked back into CM peripheral repertoires and found only in HD (HD-only) or only in T1D patients (T1D-only).

File Name: Supplementary Data 5

Description: Oligonucleotide sequences employed in the single-cell PCR studies.
